# B-cell dynamics underlying poor response upon split-inactivated influenza virus vaccination

**DOI:** 10.3389/fimmu.2024.1481910

**Published:** 2024-11-20

**Authors:** Laise Rodrigues Reis, Vanessa Silva-Moraes, Andréa Teixeira-Carvalho, Ted M. Ross

**Affiliations:** ^1^ Florida Research and Innovation Center, Cleveland Clinic, Port Saint Lucie, FL, United States; ^2^ Integrated Research Group in Biomarkers, René Rachou Institute-FIOCRUZ, Belo Horizonte, Brazil; ^3^ Department of Infection Biology, Lerner Research Institute, Cleveland Clinic, Cleveland, OH, United States; ^4^ Department of Infectious Diseases, University of Georgia, Athens, GA, United States; ^5^ Center for Vaccines and Immunology, University of Georgia, Athens, GA, United States

**Keywords:** influenza, Fluzone vaccine, humoral response, memory B-cells, adults

## Abstract

This investigation elucidated the differences in humoral and H1N1 HA-specific memory B-cells response in participants exhibiting distinct immune response patterns prior to and after vaccination with Fluzone, the quadrivalent split-inactivated seasonal influenza virus vaccine. Participants were categorized into persistent non-responders and persistent responders based on their hemagglutination-inhibition (HAI) antibody titers to the H1N1 component from each vaccine administered between the 2019-2020 to 2023-2024 seasons. Persistent responders had higher fold change in H1N1 HA-specific CD21 expressing B-cells, plasmablasts, and plasma cells. A significant increase in H1N1 HA-specific transitional B-cells in persistent non-responders was observed. The frequency and fold change of H1N1-specific IgM-expressing memory B-cells was higher in persistent non-responders. Dimensionality reduction analysis also demonstrated higher IgM expression for persistent non-responders than persistent responders. Furthermore, persistent non-responders had a significant fold change increase in IgA tissue-like memory, IgG exhausted tissue-like memory, and double negative (DN) activated memory cells. In contrast, persistent responders had increased frequency of IgG-activated memory B-cells, IgG resting B-cells and DN resting B-cells. Correlation analysis revealed a positive correlation between HAI titers and DN memory B-cells and a negative correlation between HAI titers and IgG-expressing memory B-cells in persistent non-responders. Conversely, persistent responders had a positive correlation between HAI titers and IgA resting memory B-cells and a negative correlation between IgG memory B-cells and DN memory B-cells. Overall, this study provided valuable insights into the differential immune memory B-cell responses following influenza virus vaccination and paves the way for future research to further unravel the complexities of vaccine-induced memory B-cells and ultimately improve vaccination strategies against influenza virus infection.

## 1 Introduction

Influenza remains a significant global health challenge, affecting millions of people worldwide with varying severity and resulting in thousands of deaths annually (1). The annual vaccination is the most effective measure to reduce influenza virus infection and transmission. However, vaccine efficacy varies widely among individuals, with some consistently failing to mount a protective and adequate immune response (2–4). Memory B-cells play an essential role in the adaptive immune response, involving a complex interaction between the humoral and cellular compartments aimed at inducing long-term immunity and enabling rapid response upon re-exposure to antigens (5, 6). Consequently, the differentiation and functionality of memory B-cell subsets can significantly influence the overall effectiveness of the vaccine-induced immune response.

Recent advances in cellular immunity have identified various classes of memory B-cells with distinct functions, including activated, resting, and tissue-like memory B-cells (7, 8). Activated memory B-cells function as professional antigen-presenting cells (APCs) that activate naïve T-cells (9). Resting memory B-cells, also known as classical memory B-cells, can proliferate and rapidly differentiate into plasma cells (10). Conversely, tissue-like exhausted memory B-cells display limited proliferation in response to B-cell stimuli due to elevated expression of homing and inhibitory receptors (11, 12). In addition to these classes, a subset of B-cells known as double-negative (DN) memory, characterized by the lack of expression of CD27 and IgD markers, has been identified. As observed with tissue-like exhausted B-cell memory, DN B-cell memory has been described as a premature exhaustion stage in infectious diseases, expressing inhibitory receptors, poor proliferation, and poor antibody responses (13–15).

Since several memory B-cell subsets have distinct roles during the immune response, understanding the differentiation and dynamics between humoral and cellular responses can provide valuable insights into the mechanism underpinning sustained immune protection, vaccine strategies, and outcomes. The present investigation aimed to elucidate the differences in the humoral responses and H1N1 HA-specific memory B-cells between participants with distinct immune response patterns prior to and after vaccination with the split-inactivated quadrivalent seasonal influenza virus vaccine (Fluzone, Sanofi-Pasteur). The findings provided deeper insights into the dynamics of B-cell responses, contributing to the growing knowledge on immune responses following split-inactivated virus vaccination in adults.

## 2 Materials and methods

### 2.1 Study population and design

**Figure 1:**
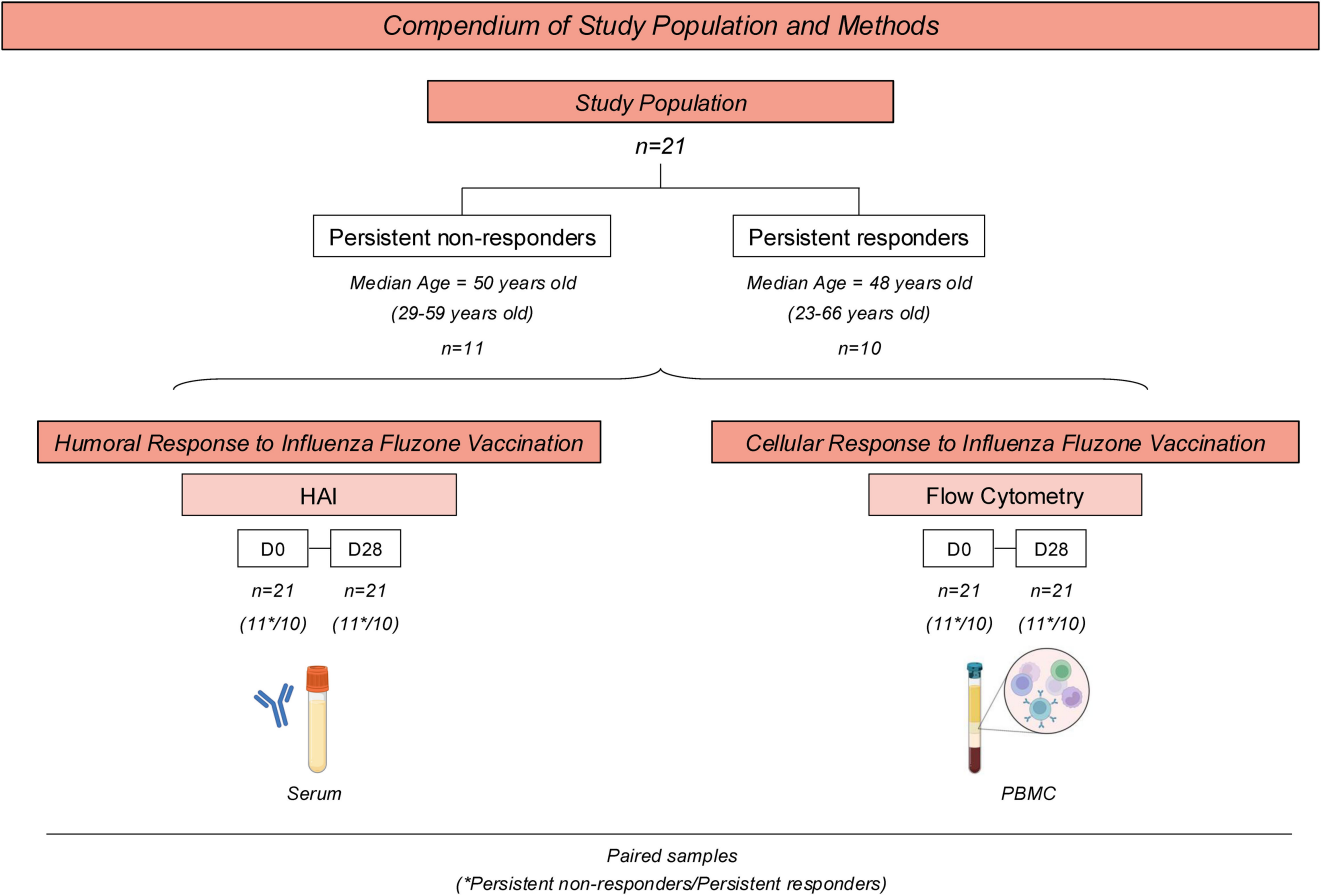
Compendium of Study Population and Methods for analyzing H1N1 HA-specific humoral and cellular response upon Fluzone Influenza vaccination in adults. The study comprises 21 healthy adults vaccinated with the standard dose of Fluzone Quadrivalent Seasonal Influenza vaccine. Participants were categorized into two subgroups based on their influenza hemagglutination-inhibition (HAI) antibody titers to the H1N1 seasonal strains used in the vaccine each season between 2019-2020 to 2023-2024. Persistent non-responders (n=11) were classified as participants who did not have a seroprotective HAI titer ≥1:40 post-vaccination nor did they not have a 4-fold or greater rise in HAI titer (seroconversion) between pre-vaccination and post-vaccination each season. Persistent responders (n=10) were classified as participants who both seroconverted and had a seroprotective HAI titer for a minimum of three years of following the initial vaccination. Each subgroup’s median age and ranges are provided below the respective subgroup boxes. The humoral response was assessed using HAI and ELISA at day 0 (D0) and 28 days (D28) after vaccination. Flow Cytometry was performed to determine H1N1 HA-specific cellular response before (D0) and 28 days (D28) after immunization.

Participants were recruited between September and March of each influenza season between 2021-2022 and 2023-2024 in Athens, Georgia, USA. All participants were enrolled with written informed consent and the research protocol was approved by the Institutional Review Board from the University of Georgia (IRB #20224877). **Figure 1** summarizes the study population, design, and methods.

The study population comprised 21 healthy adults between the ages of 23 and 66 years old (**Table 1**) who had not been vaccinated against seasonal influenza viruses. Participants were vaccinated with the standard dose of Fluzone quadrivalent seasonal influenza vaccine (Sanofi Pasteur, Swiftwater, PA, USA). Vaccine-induced humoral and cellular responses to the H1N1 component of the influenza virus vaccine were evaluated.

**Table 1:** Demographic data of the study population.

	Persistent non-responders (N, %)	Persistent responders (N, %)
No. of individuals, N (%)	11 (52.4%)	10 (47.6%)
Age, y	Median = 50	Median = 48
	Range = 29-59	Range = 23-66
Male	4 (36.4%)	3 (30.0%)
Female	7 (63.6%)	7 (70.0%)
White: non-Hispanic/Latino	10 (90.9%)	9 (90.0%)
White: Hispanic/Latino	1 (9.1%)	0 (0.0%)
Black: non-Hispanic/Latino	0 (0.0%)	1 (10.0%)
Comorbidities	0 (0.0%)	0 (0.0%)

### 2.2 Biological samples

Each season, blood samples were collected from all participants before vaccination (day 0; D0) and 28 days (D28) after vaccination with the standard dose of Fluzone quadrivalent seasonal influenza vaccine (Sanofi Pasteur, Swiftwater, PA, USA). The vaccine included four influenza virus strains recommended for the Northern Hemisphere influenza season of 2021-2022 (A/Victoria/2570/2019 - H1N1; A/Cambodia/e0826360/2020 - H3N2; B/Washington/02/2019 - B/Victoria lineage; B/Phuket/3073/2013 - B/Yamagata lineage), 2022-2023 (A/Victoria/2570/2019 -H1N1; A/Darwin/9/2021 - H3N2; B/Austria/1359417/2021 - B/Victoria lineage; B/Phuket/3073/2013 - B/Yamagata lineage) and 2023-2024 (A/Victoria/4897/2022 - H1N1; A/Darwin/9/2021 - H3N2; B/Austria/1359417/2021 - B/Victoria lineage; B/Phuket/3073/2013 - B/Yamagata lineage).

Samples were collected in vacutainer serum separation tubes (SST) (BD Biosciences, Franklin Lakes, NJ, USA) and centrifuged at 1,000 x g for 10 min at RT. After centrifugation, the serum was isolated from the gel layer and red blood cell pellet. Additional samples were collected in vacutainer K2 EDTA tubes (BD Biosciences, Franklin Lakes, NJ, USA), and samples were homogenized and transferred to vacutainer cell preparation tubes (CPT) (BD Biosciences, Franklin Lakes, NJ, USA) for peripheral blood mononuclear cells (PBMC) isolation. The tubes were centrifugated at 1,800 x g for 20 min at RT. Sera were removed and PBMCs were washed in 1x Phosphate-buffered saline (PBS). Residual red blood cells were removed by incubation with ACK red blood cell (RBC) lysis buffer (Life Technologies, Carlsbad, CA, USA). After additional washes, cells were resuspended in cold freezing media (90% Bovine Serum with 10% DMSO) to a final concentration of 1x107 cells/mL. Cells were stored in liquid nitrogen until use for *in vitro* B-cell differentiation and subsequent flow cytometry assay.

### 2.3 Influenza Hemagglutination-inhibition assay

Influenza Hemagglutination-inhibition (HAI) assays were performed to quantify HA-specific antibodies by measuring the inhibition of influenza virus binding to sialic acid on erythrocytes. The assay was carried out as previously described by the WHO Manual for the Laboratory Diagnosis of Influenza (16). Briefly, serum samples were diluted with receptor-destroying enzyme (RDE) (Denka Seiken, Co., Japan) at a 1:3 ratio and incubated overnight at 37°C. After incubation, the RDE was inactivated by placing the serum-RDE mixture in a 56°C water bath for 45 minutes. Subsequently, six volumes of PBS were added to the mixture and diluted two-fold in V-bottom 96-well microtiter plates. Turkey red blood cells (TRBC) (Lampire Biologicals, Pipersville, PA, USA) were diluted in PBS to a concentration of 0.8%, and the H1N1 virus was adjusted to a concentration of eight hemagglutination units (HAU)/50μL. An equal volume of the prepared virus was added to the V-bottom microtiter plates. Plates were covered and incubated for 20 minutes at room temperature, followed by the addition of 0.8% TRBC to each well. The plates were gently mixed and incubated for 30 min at RT. HAI titers were determined by the reciprocal dilution of the last well that was not observed RBC agglutination. Seroprotection was classified as HAI titers ≥1:40 and seroconversion was classified as a 4-fold or greater increase in the ≥1:40 HAI titer.

### 2.4 B-cell Immunophenotyping by flow cytometry

PBMC samples were used to assess the H1N1 HA-specific B-cell responses and were processed as previously described (17–20) with modifications. Briefly, PBMC stored in liquid nitrogen were thawed in a water bath at 37°C and resuspended in 10mL of warm supplemented Roswell Park Memorial Institute (RPMI) 1640 Medium (10% heat-inactivated AB normal human serum; 1% penicillin/streptomycin and 2mM L-glutamine) with 25 U/mL of benzonase (Millipore Sigma, Burlington, MA, USA). Cells were submitted to centrifugation at 500 x g for 7 min at 4oC. After centrifugation, the supernatant was removed, and cells were washed with 10mL of supplemented RPMI-1640 media without benzonase. Subsequently, the supernatant was removed, cells were resuspended in 1mL of supplemented RPMI-1640 and counted in the LUNA-II™ Automated Cell Counter (Logos Biosystems, South Korea) to adjust to a final concentration of 1.0 x 10^6^/mL. Cells were transferred to 48-well suspension plates (1mL/well) and submitted to *in vitro* differentiation by adding 10μL/10mL of a human B-poly-S polyclonal B-cell stimulator (ImmunoSpot, Shaker Heights, OH, USA) in order to amplify the signal of rare populations of B cells prior to subsequent analyses. The plates were incubated for seven days at 37°C in 5% CO_2_.

After seven days of incubation, cells were gently mixed, transferred to a 15mL tube, and centrifuged at 500 x g for 5 min at 4°C. After centrifugation, the supernatant was removed, cells were resuspended in 1mL of supplemented RPMI-1640 and counted in the LUNA-II™ Automated Cell Counter to adjust to a final concentration of 1.0 x 106/200μL/mL. Cells were transferred to a 96-well plate (200μL/well) and treated with protein transport inhibitor cocktail (Invitrogen, Waltham, MA, USA) for four hours at 37oC in 5% CO2. Cells were washed with PBS containing 2mM ethylenediamine-tetra-acetic acid (EDTA), resuspended in human Fc-blocking solution (BD, Franklin Lakes, NJ, USA) and incubated for 10 min at RT protected from light. Cells were washed with 1x PBS and stained for 30 min at 4oC with HA-derived recombinant H1N1 (A/Wisconsin/588/2019) probe conjugated to streptavidin-PE. Next, cells were rewashed and incubated with a mix of monoclonal antibodies (mAbs) to cell surface staining, comprising of anti-CD3/OKT3/Alexa-Fluor 700; anti-CD14/M5E2/Alexa-Fluor 700; anti-CD19/HIB19/APC-Fire 750, anti-CD20/2H7/BV605, anti-CD21/B-ly4/BV480, anti-CD24/ML5/PE-Cy5, anti-CD27/M-T271/PE-Cy7, anti-CD38/HIT2/PE-Dazzle 594, anti-CD85j/GHI-75/Alexa-Fluor 647, anti-CXCR3/1C6-CXCR3/BB700, anti-IgD/IA6-2/BV421, anti-IgM/G20-127/FITC, anti-IgA/IS11-21E11/PerCP-Vio700 and anti-IgG/G18-145/BV510. Stained cells were washed twice with FACS buffer and resuspended in fixation/permeabilization solution (BD, Franklin Lakes, NJ) for fixation. Cells were washed twice with Perm/Wash Buffer (BD, Franklin Lakes, NJ) and incubated with a mix of monoclonal antibodies (mAbs) to cell intracellular staining, including IL-4/MP4-25D2/BV711; IL-6/MQ2-13A5/BB515; IL-10/JES3-9D7/BV650 and TNF-a/Mab11/BV785. Cells were washed with Perm/Wash Buffer once and with FACS Buffer twice before data acquisition. All samples were acquired on an Aurora Spectral Flow Cytometer system with four lasers (Cytek, Fremont, CA, USA).

### 2.5 Data analysis

Flow cytometry data were analyzed in FlowJo 10.10.0 (BD Biosciences, Franklin Lakes, NJ, USA) (**Supplementary Figure S1**) and statistical analysis was performed in GraphPad Prism 10.2.3 (Dotmatics, Boston, MA, USA). The fold change was determined by the results observed at D28 after vaccination divided by those detected before vaccination at D0. Comparative analysis between the Persistent non-responder and Persistent responders was carried out by Student t-test. In all cases, significant differences were considered when p ≤ 0.05. High-dimensional analysis was performed in FlowJo 10.10.0 to generate t-Distributed Stochastic Neighbor Embedding (t-SNE) plots. The analysis of memory B-cell subsets was performed using a supervised selection strategy. A total of 5,000 iterations were performed using CD19, CD20, CD21, CD27, IgD, IgM, IgA and IgG parameters and manually assigned to the markers IgM, IgA and IgG. Correlation analysis was performed using a Spearman rank correlation coefficient between two ranked variables. Heatmaps were constructed to represent the strength of positive or negative correlation, and in all cases, significant data were considered when p ≤ 0.05.

## 3 Results

### 3.1 Study population and serological status

**Figure 2:**
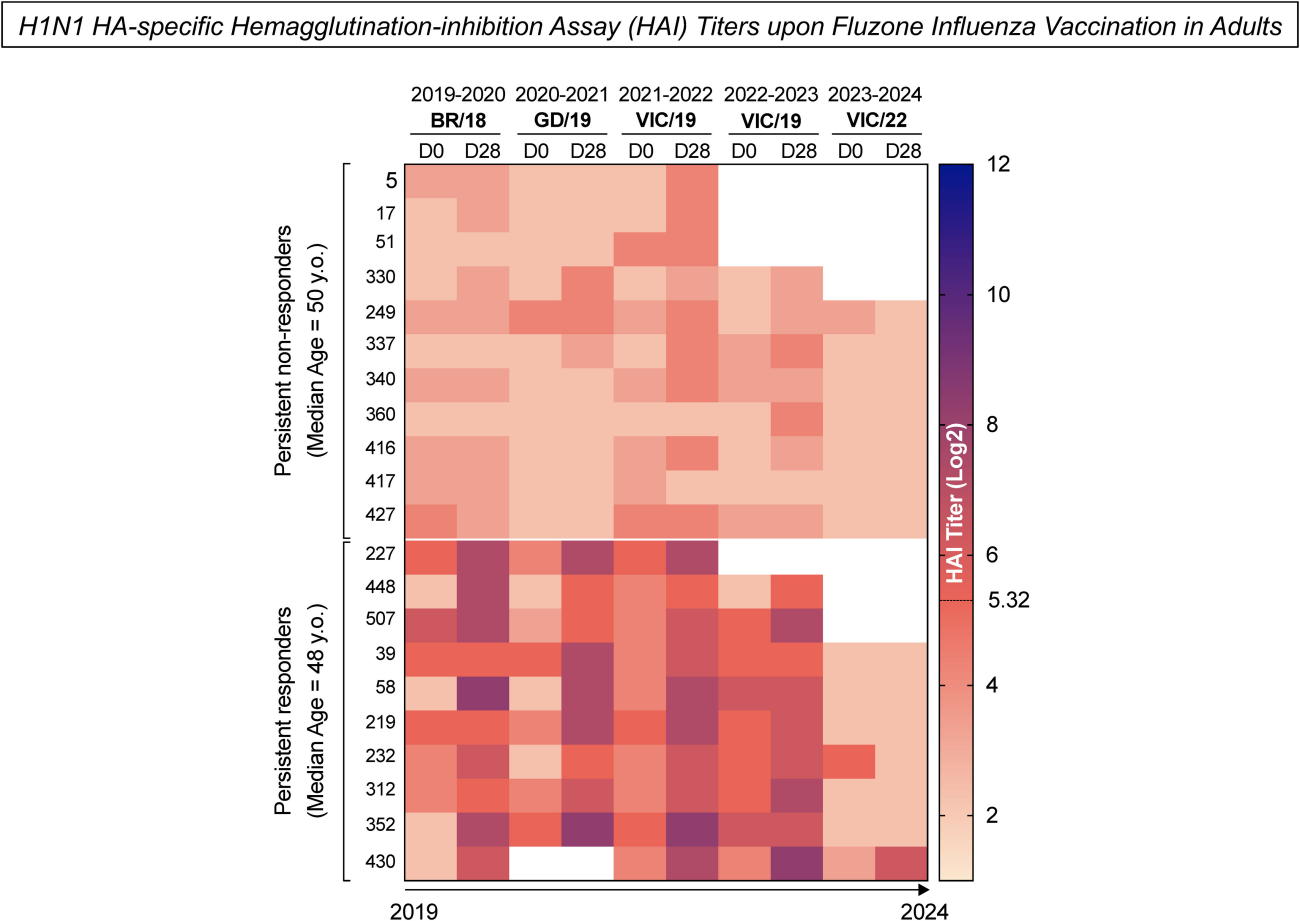
H1N1 HA-specific Hemagglutination-inhibition Assay (HAI) titers upon Fluzone Influenza vaccination in adults. The heatmap represents the H1N1 HA-specific HAI titers measured from UGA4 (2019–2020) to UGA8 (2023-2024) at day 0 (D0) and 28 days (D28) after Fluzone Influenza vaccination in adults. The columns denote the H1N1 HA-specific HAI titers measured at two time points (D0 and D28) from UGA4 (2019-2020) to UGA8 (2023-2024). The rows represent the two study subgroups: persistent non-responders (median age = 50 y.o.) – participants who did not have a seroprotective HAI titer Log2 ≥5.32 post-vaccination or did not have a 4-fold or greater rise in HAI titer (seroconversion) between pre-vaccination and post-vaccination each season; and persistent responders (median age = 48 y.o.) - participants who both seroconverted and had a seroprotective HAI titer for a minimum of three years of following the initial vaccination. Color gradients denote the levels of HAI titers, with lighter shades of pink representing lower titers and darker shades of blue indicating higher titers.

Healthy adults (n=21) between the ages of 23 and 66 years old were selected for B-cell analysis after vaccination with the standard dose of Fluzone quadrivalent seasonal split-inactivated influenza virus vaccine. Participants were categorized into two subgroups based on their influenza hemagglutination-inhibition (HAI) antibody titers to the H1N1 seasonal strains used in the vaccine each season between 2019-2020 to 2023-2024. Persistent non-responders (n=11) were classified as participants who did not have a seroprotective HAI titer ≥1:40 post-vaccination nor did they not have a 4-fold or greater rise in HAI titer (seroconversion) between pre-vaccination at day 0 (D0) and post-vaccination at day 28 (D28) each season. Persistent responders (n=10) were classified as participants who both seroconverted and had a seroprotective HAI titer for at least three years following the initial vaccination (**Figure 2**).

### 3.2 Phenotypic features of H1N1 HA-specific B-cells upon Fluzone influenza vaccination

**Figure 3:**
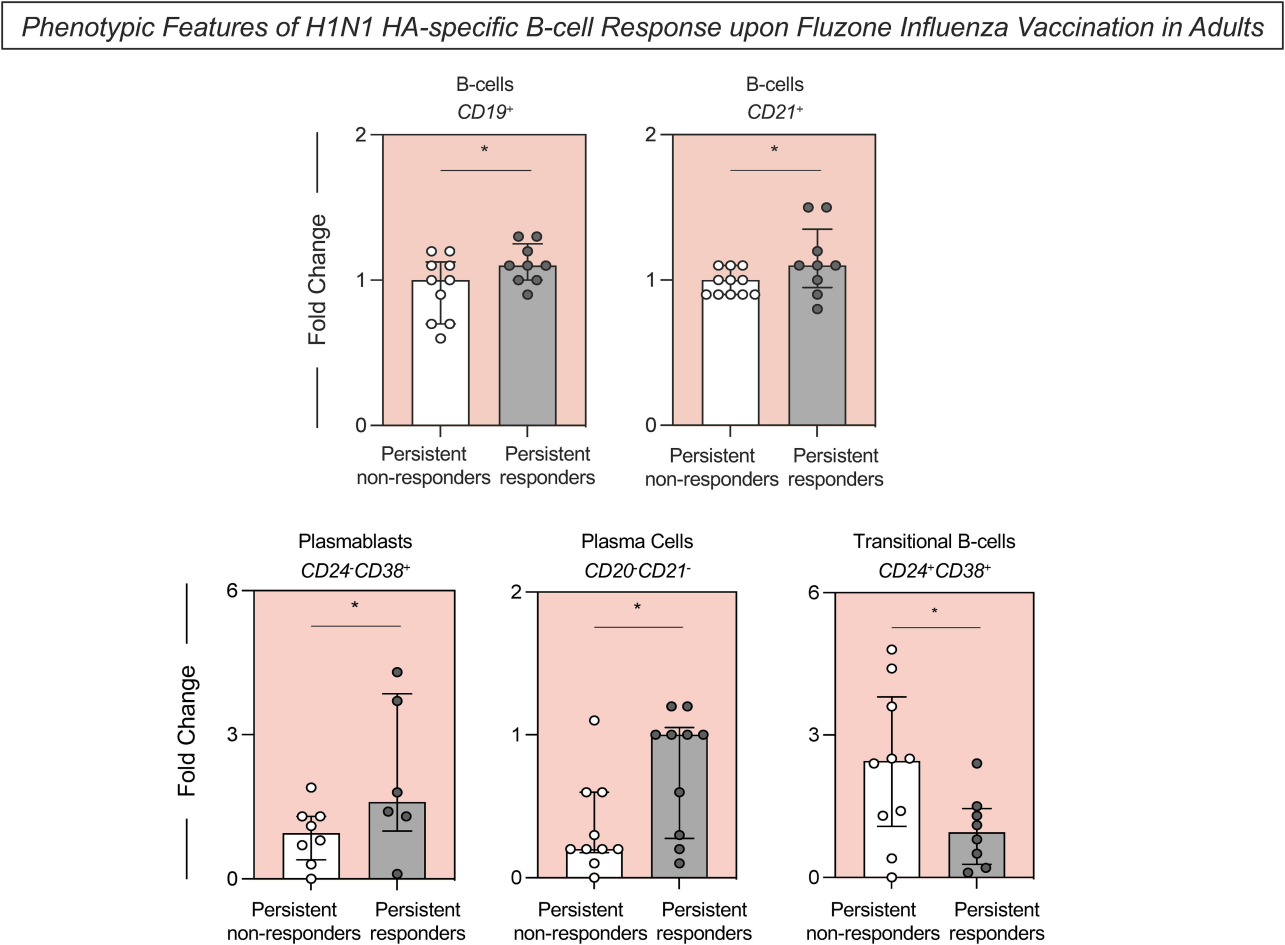
Phenotypic features of H1N1 HA-specific B-cell response upon Fluzone Influenza vaccination in adults. Analysis of H1N1 HA-specific B-cell subsets (B-cells–CD19^+^; B-cells–CD21^+^; Plasmablasts–CD24^-^CD38^+^; Plasma Cells–CD20^-^CD21^-^ and Transitional B-cells–CD4^+^CD38^+^) was carried out by Flow Cytometry, as described in Materials and Methods. The H1N1 HA-specific B-cell response was evaluated in persistent non-responders (white bars) and persistent responders (grey bars), and data are displayed as a scattering distribution of individual values over bars expressed as fold change, determined by the values observed at D28 divided by those detected at D0. A comparative analysis between groups was performed using the Student t-test. Significant differences were considered at p ≤ 0.05 and represented by ‘*’.

H1N1 HA-specific B-cell populations were characterized using surface markers to define H1N1 HA-specific total B-cells (H1N1^+^CD19^+^), B-cells expressing CD21 (H1N1^+^CD21^+^), plasmablasts (H1N1^+^CD24^-^CD38^+^), plasma cells (H1N1^+^CD20^-^CD21^-^) and transitional B-cells (H1N1^+^CD24^+^CD38^+^) (**Figure 3**). Persistent responders had significantly higher H1N1 HA-specific total B-cell fold changes following vaccination than persistent non-responders. These included B-cells that expressed cell surface CD21, plasmablasts (CD24^-^CD38^+^) and plasma cells (CD20^-^CD21^-^) compared to the persistent non-responders (**Figure 3**). Conversely, there was a significant increase in H1N1 HA-specific transitional B-cells (CD24^+^CD38^+^) in persistent non-responders compared to persistent responders. A range of additional B-cell phenotypes and B-cell cytokines was evaluated but remained unaltered between these two groups (**Supplementary Figure S2**).

### 3.3 H1N1 HA-specific IgM, IgA and IgG B-cell memory responses upon Fluzone influenza vaccination

**Figure 4:**
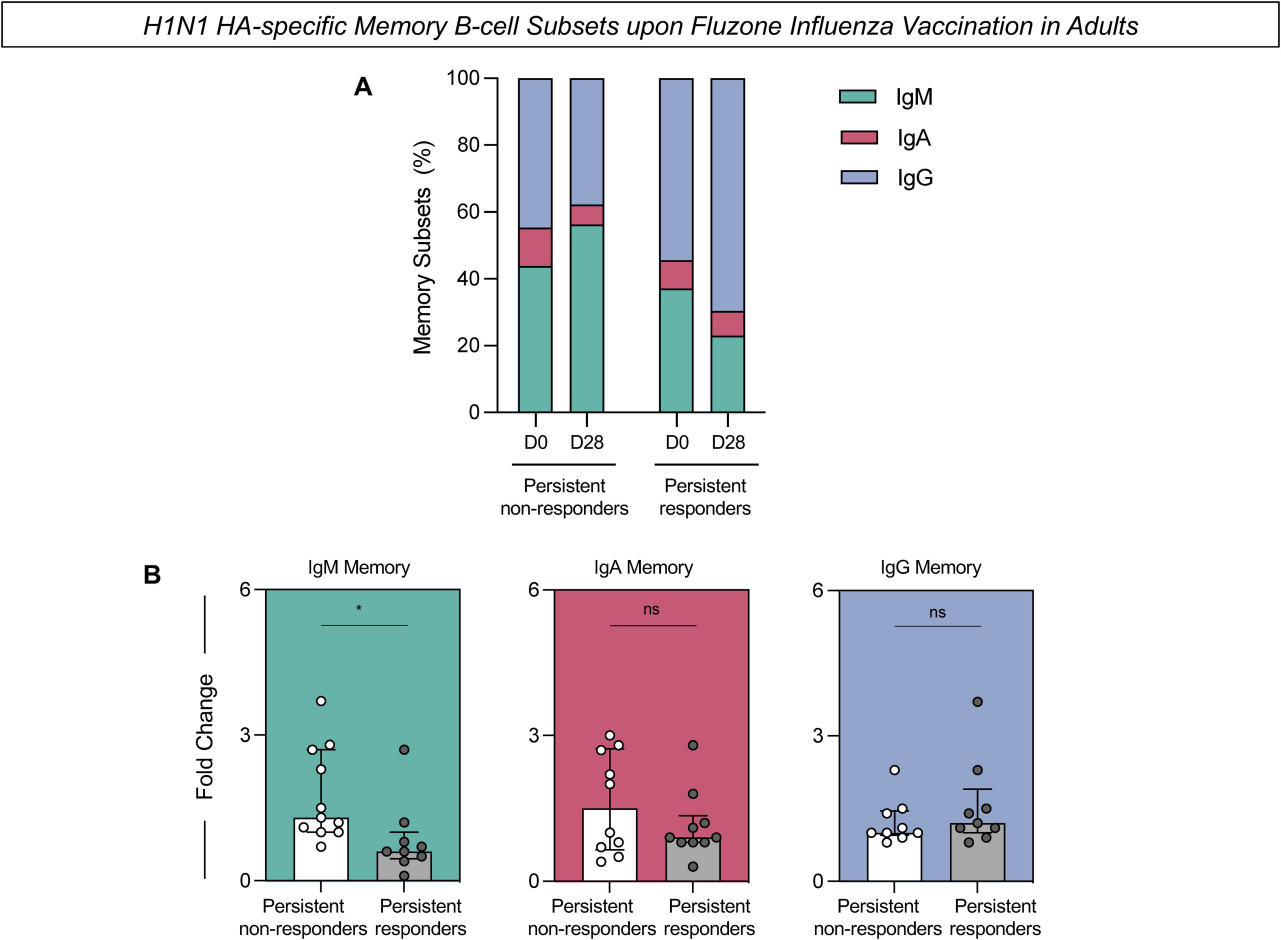
H1N1 HA-specific memory B-cell subsets upon Fluzone Influenza vaccination in adults. Analysis of H1N1 HA-specific memory B-cell subsets (IgM memory–IgD^+^IgM^+^; IgA memory–IgD^-^IgA^+^ and IgG memory–IgD^-^IgG^+^) was carried out by Flow Cytometry as described in Materials and Methods. **(A)** The percentage of memory subsets at days 0 (D0) and 28 (D28) after Fluzone Influenza vaccination in persistent non-responders and persistent responders subgroups. **(B)** H1N1 HA-specific B-cell response evaluated in persistent non-responders (white bars) and persistent responders (grey bars) displayed as a scattering distribution of individual values over bars expressed as fold change, determined by the values observed at D28 divided by those detected at D0. A comparative analysis between groups was performed using the Student t-test. Significant differences were considered at p ≤ 0.05 and represented by ‘*’. Non-significant results are represented by ‘ns’.

PBMCs were assessed for the expression of H1N1 HA-specific memory B-cells before and after vaccination (**Figure 4**). The frequency of H1N1 HA-specific IgM-expressing memory cells was consistently higher in the persistent non-responders at D0 and D28 compared to the persistent responders (**Figure 4A**). Additionally, there was a significant increase in the fold change of H1N1 HA-specific IgM in the persistent non-responders compared to persistent responders (**Figure 4B**). There were no statistically significant differences in the fold change of IgA or IgG expressing PBMCs collected from the two groups.

**Figure 5:**
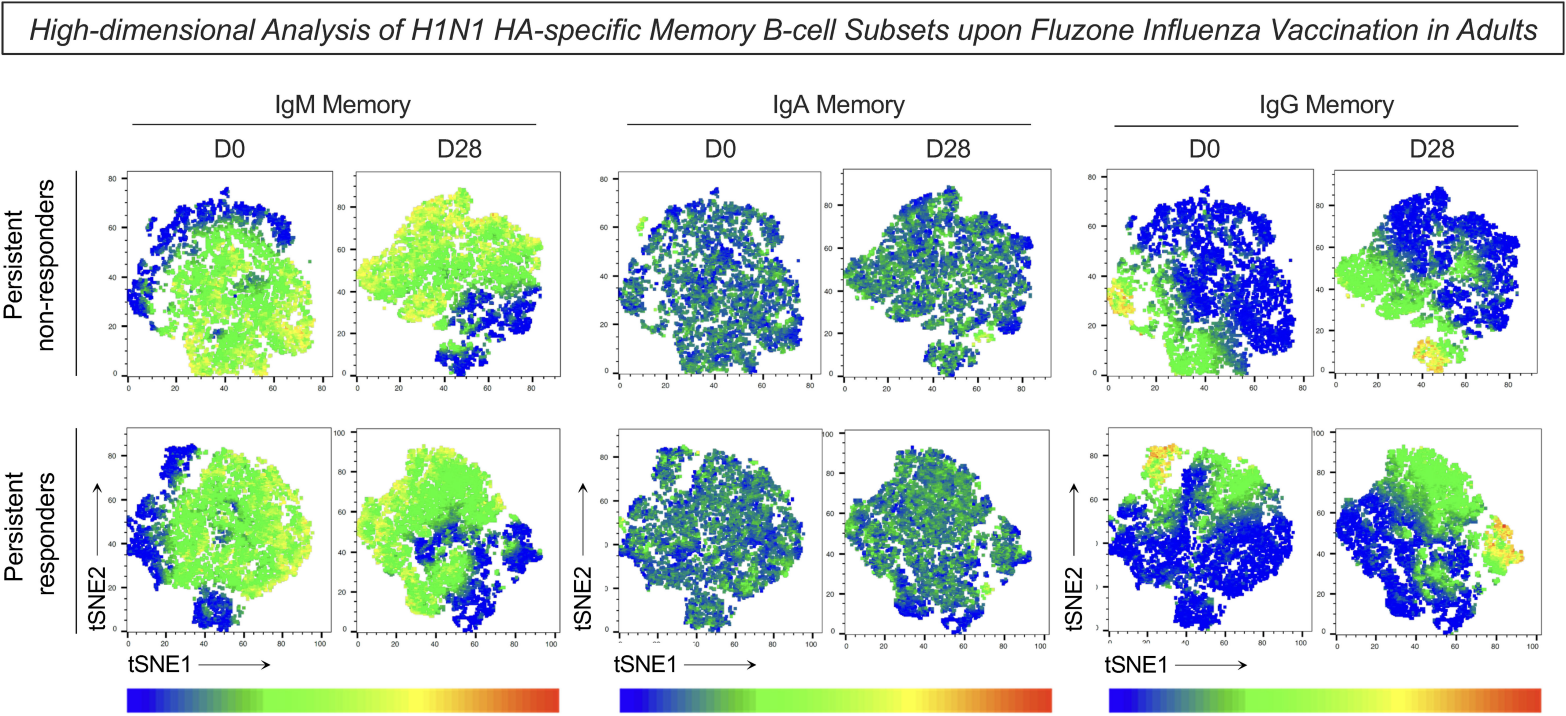
High-dimensional analysis of H1N1 HA-specific memory B-cell subsets upon Fluzone Influenza vaccination in adults. Analysis of H1N1 HA-specific memory B-cell subsets (IgM memory–IgD^+^IgM^+^; IgA memory–IgD^-^IgA^+^ and IgG memory–IgD^-^IgG^+^) was assessed by Flow Cytometry as described in Materials and Methods. Dimensionality reduction data was carried out using t-SNE (t-Distributed Stochastic Neighbor Embedding) to visualize high-dimensional data of H1N1 HA-specific memory B-cell subsets. A total of 5,000 iterations were performed using CD19, CD20, CD21, CD27, IgD, IgM, IgA and IgG parameters and manually assigned to the markers IgM, IgA and IgG. The color gradient represents the cell surface expression level of the markers, with dark red referring to high expression and dark blue to low expression.

Complementary analysis of the H1N1 HA-specific memory B-cells was performed using t-SNE to visualize high-dimensional data of memory B-cell subsets (**Figure 5**). Dimensionality reduction of flow cytometry data further illustrated higher expression of IgM in the persistent non-responders compared to persistent responders at D0 and D28 after influenza virus vaccination (**Figure 5**). No difference was observed in the expression of IgA and IgG between groups at either timepoint.

### 3.4 H1N1 HA-specific B-cell memory subsets in Fluzone influenza-vaccinated participants

**Figure 6:**
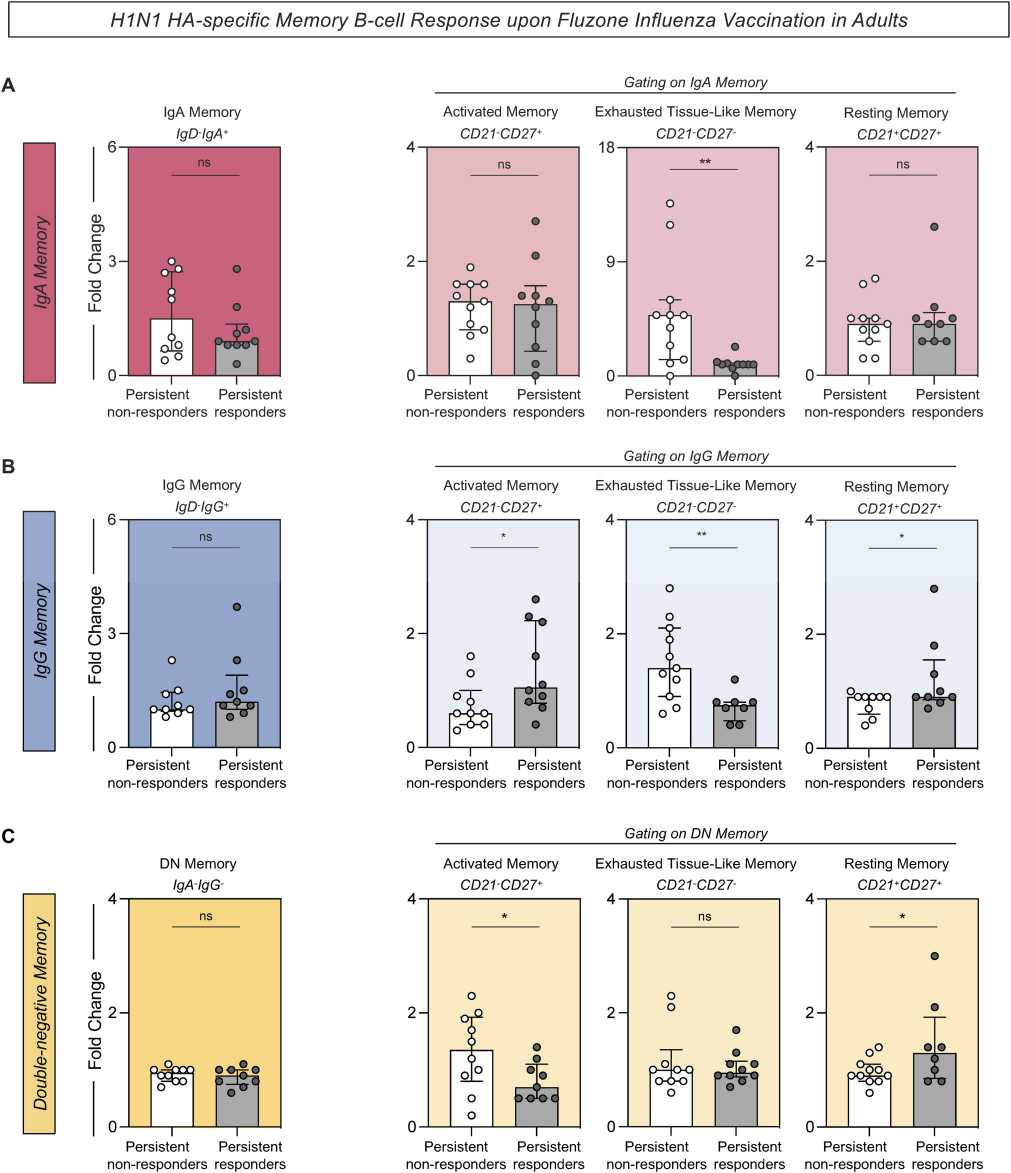
H1N1 HA-specific memory B-cell response upon Fluzone Influenza Vaccination in adults. Analysis of H1N1 HA-specific memory B-cell was carried out by Flow Cytometry as described in Materials and Methods. **(A)** H1N1 HA-specific IgA memory (IgD^-^IgA^+^), IgA activated memory (IgA^+^CD21^-^CD27^+^), IgA exhausted tissue-like memory (IgA^+^CD21^-^CD27^-^) and IgA resting memory (IgA^+^CD21^+^CD27^+^). **(B)** H1N1 HA-specific IgG memory (IgD^-^IgG^+^), IgG activated memory (IgG^+^CD21^-^CD27^+^), IgG exhausted tissue-like memory (IgG^+^CD21^-^CD27^-^) and IgG resting memory (IgG^+^CD21^+^CD27^+^). **(C)** H1N1 HA-specific DN memory (IgA^-^IgG^-^), DN activated memory (IgA^-^IgG^-^CD21^-^CD27^+^), DN exhausted tissue-like memory (IgA^-^IgG^-^CD21^-^CD27^-^) and DN resting memory (IgA^-^IgG^-^CD21^+^CD27^+^). The H1N1 HA-specific B-cell response was evaluated in persistent non-responders (white bars) and persistent responders (grey bars), and data are displayed as a scattering distribution of individual values over bars expressed as fold change, determined by the values observed at D28 divided by those detected at D0. A comparative analysis between groups was performed using the Student t-test. Significant differences were considered at p ≤ 0.05 and represented by ‘*’. Non-significant results are represented by ‘ns’.

H1N1 HA-specific memory B-cells were evaluated from persistent non-responders and persistent responders before and after vaccination. There were no significant differences in the overall levels of memory cells expressing either IgA or IgG, as well as double-negative (DN) memory B-cells (IgD^-^CD27^-^IgA^-^IgG^-^) between the persistent non-responders and persistent responders. However, there were variations in specific subsets of these memory B-cells including a significant fold change increase in the IgA exhausted tissue-like memory cells (IgA^+^CD21^-^CD27^-^) (**Figure 6A**), IgG exhausted tissue-like memory (IgG^+^CD21^-^CD27^-^) (**Figure 6B**), and DN activated memory cells in persistent non-responders compared to persistent responders (**Figure 6C**). Conversely, there were increased levels of IgG activated memory B-cells (IgG^+^CD21^-^CD27^+^), IgG resting memory B-cells (IgG^+^CD21^+^CD27^+^) (**Figure 6B**), and DN resting memory B-cells (IgA^-^IgG^-^CD21^+^CD27^+^) (**Figure 6C**) in the persistent non-responders compared to persistent responders.

### 3.5 Correlation between H1N1 HA-specific humoral and cellular memory response following influenza virus vaccination

**Figure 7:**
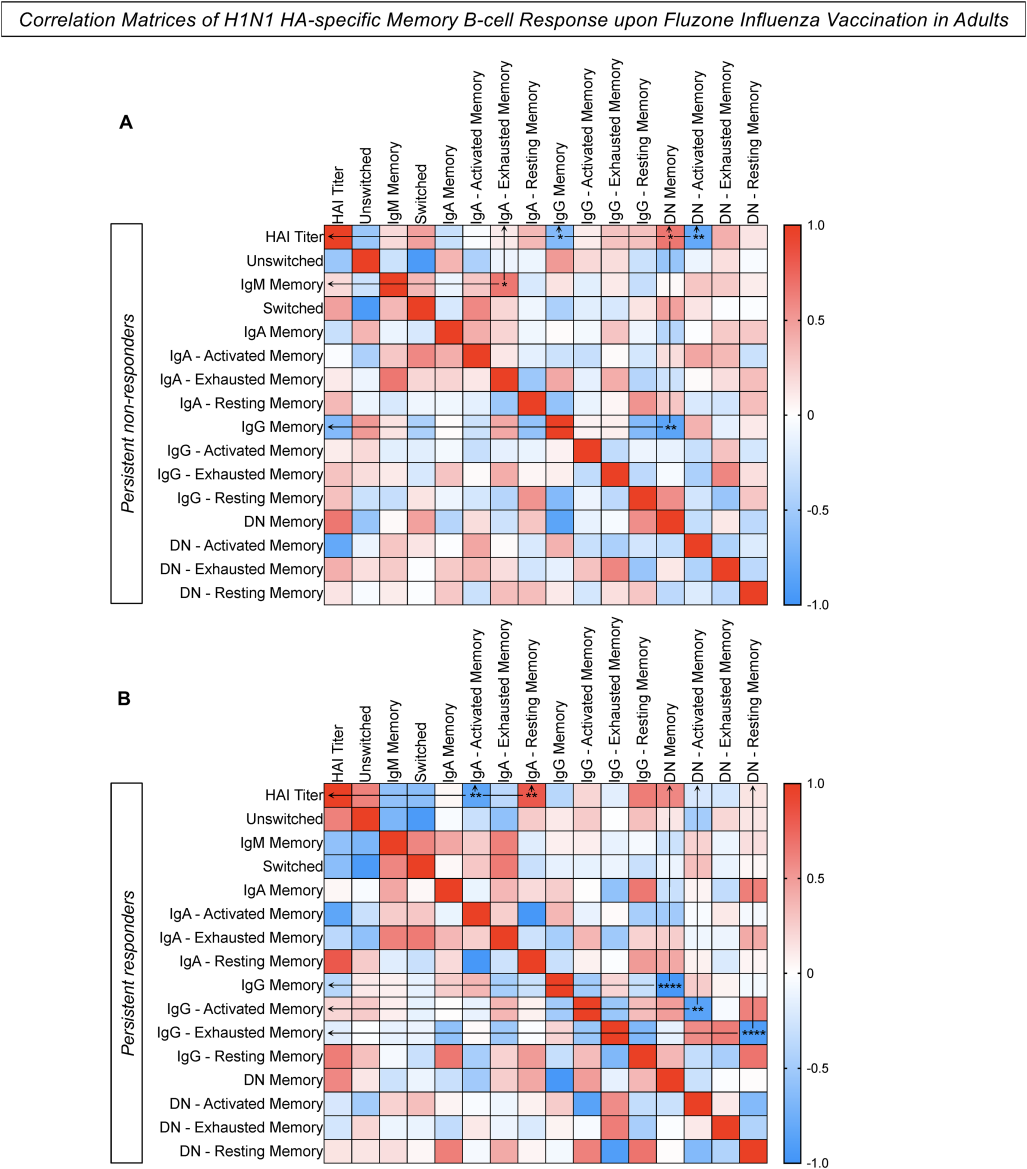
Correlation Matrices of H1N1 HA-specific memory B-cell response upon Fluzone Influenza Vaccination in adults. Correlation analysis between humoral and memory B-cell response was performed for the groups **(A)** Persistent non-responders and **(B)** Persistent responders using a Spearman rank correlation coefficient. The color gradient represents the strength of positive (red) or negative (blue) correlation between two ranked variables. Only significant data are indicated. * p ≤ 0.05, ** p ≤ 0.01 and **** p ≤ 0.0001.

Correlation analysis was performed to assemble correlogram matrices to define connections between H1N1 HA-specific humoral and cellular memory responses following vaccination. Data analysis for the persistent non-responders showed a significant positive correlation between “HAI titers/DN memory B-cells”, and “IgM memory/IgA exhausted memory B-cells” (**Figure 7A**). Moreover, negative correlations were observed for “HAI titers/IgG memory B-cells”, “HAI titers/DN activated memory B-cells”, and “IgG memory/DN memory B-cells” for persistent non-responders. In contrast, persistent responders had a significant positive correlation between “HAI titers/IgA resting memory B-cells” and strong negative correlations for “HAI titers/IgA activated memory B-cells”, “IgG memory/DN memory B-cells”, “IgG activated memory/DN activated memory B-cells”, and “IgG exhausted memory/DN resting memory B-cells” (**Figure 7B**).

## 4 Discussion

The immune response to influenza virus vaccination involves complex interactions between the humoral and cellular immune compartments to induce a robust and long-lasting immune memory. Following vaccination, B-cells undergo activation, differentiation, and maturation to produce specific and long-term antibodies that significantly prevent viral infection (14, 21, 22). The dynamic of the B-cell response is essential for understanding the variability in influenza virus vaccine responsiveness among individuals and subsequently designing methods to enhance vaccine performance. This study assessed the phenotypic and functional profile of H1N1 HA-specific B-cell response among participants who presented distinct immune response patterns prior to and after vaccination with the split-inactivated quadrivalent seasonal influenza virus vaccine (Fluzone, Sanofi-Pasteur). Additionally, correlations between humoral and B-cell memory responses were explored to gain deeper insights into effective long-term immunity.

Persistent responders had significantly higher fold change in H1N1 HA-specific total B-cells following vaccination than persistent non-responders, including B-cells that expressed cell surface CD21, plasmablasts, and plasma cells. The CD21 cell surface marker is important for distinguishing the stages of B-cell development, with B-cells expressing low levels of CD21 characterized as a less mature population (23, 24). Moreover, this B-cell population expresses multiple inhibitory receptors and responds poorly to B-cell receptor (BCR) stimulation (24). Lacking the CD21 B-cell population is associated with constant antigen exposure and B-cell activation (15, 25–28). Additionally, there was a significant increase in H1N1 HA-specific transitional B-cells in persistent non-responders compared to persistent responders. Transitional B-cells denote a stage between immature and mature peripheral B-cells expressing higher levels of IgM and exhibiting less proliferation and differentiation than mature B-cells (23, 29–31). Furthermore, only a few transitional B-cells enter the mature and long-lived B-cell compartment due to the self-reactive characteristic, leading to an unresponsive state or cell death (32–34). Altogether, in this study, persistent non-responders have a higher prevalence of immature B-cells, indicating a potential impairment in the maturation and development of H1N1 HA-specific B-cells in these individuals. Consequently, this results in lower proliferation and differentiation of B cells into plasmablasts and plasma cells, which are responsible for producing immunoglobulins.

The frequency of H1N1 HA-specific IgM-expressing memory B-cells was consistently higher in persistent non-responders, both prior to and post-vaccination compared to persistent responders (**Figure 4A**). In addition, there was a significant increase in the fold change for this memory B-cell subset in persistent non-responders, as well as higher IgM expression as visualized in dimensionality reduction analysis (**Figure 4B**). The increased IgM-expressing memory B-cells in persistent non-responders could underscore the potential impairment in the B-cell class switch recombination (CSR). CSR is essential for effectively transitioning from IgM to other immunoglobulin classes, such as IgA and IgG, contributing to an effective and long-lasting immune response (35, 36). This may reflect an initial immune response activation in persistent non-responders that fails to progress to a more mature and effective response.

Due to the importance of memory B-cells in generating a more robust antibody-mediated responses, distinct B-cell memory subsets were evaluated between groups. Although no significant differences were observed in the levels of IgA, IgG, and DN memory B-cells levels between persistent responders and persistent non-responders, specific memory B-cell subsets had notable variations. Persistent non-responders had a significant fold change increase in the IgA tissue-like memory B-cells, IgG exhausted tissue-like, and DN-activated memory B-cells. These results highlight that the memory B-cell responses following vaccination in persistent non-responders are predominantly driven by B-cell memory subsets exhibiting function impairment and failure mount a proper antibody-mediated response effectively. Exhausted B-cells are associated with chronic infections and autoimmune diseases, where continuous antigen stimulation leads to functional impairment (12, 14, 15, 26). Given the frequent exposure of individuals to influenza viruses through infection or vaccination, this observation may imply that repeated antigenic stimulation could drive B-cells towards an exhausted phenotype, thereby compromising their functional properties and survival. Conversely, persistent responders had increased IgG-activated memory B-cells, IgG resting B-cells and DN resting B-cells. The presence of activated and resting memory B-cell subsets indicates a more robust, rapid, and effective secondary immune response, underscoring the efficient development of long-term immunity in these individuals.

The correlation analysis of H1N1 HA-specific humoral and cellular memory B-cell responses revealed a significant positive correlation between HAI titers and DN memory B-cells and a negative correlation between HAI titers and IgG memory B-cells in persistent non-responders. These findings suggest that the HAI titers are not associated with a robust IgG memory in persistent non-responders, but rather with DN memory B-cells. This supports the hypothesis that the immune response in this group is predominantly characterized by an exhausted memory B-cell phenotype, leading to less effective memory and impairment in the generation and maintenance of the antibody-mediated responses. Overall, this could be a key factor in the failure of vaccines to elicit protective immunity following influenza virus vaccination in persistent non-responders.

In contrast, persistent responders had different correlation patterns indicating a more effective immune response to the vaccine. The significant positive correlation between HAI titers and IgA resting memory B-cells indicates that IgA resting memory plays an essential role in maintaining the humoral response by rapidly reactivating upon antigen exposure and contributing to sustaining antibody production (37). Furthermore, the strong negative correlation between IgG memory B-cells and DN memory B-cells reinforces that the memory B-cell response in persistent non-responders is predominantly mediated by IgG memory, which is essential for the effective development of long-term immunity.

The distinct immune response patterns observed between the two groups highlight the complexity of the immune memory B-cell landscape in response to influenza virus vaccination. These findings underscore the importance of a balanced and well-regulated memory B-cell response for effective vaccine-induced immunity. Moreover, understanding the factors contributing to the persistence of non-responsiveness in certain people could inform the development of targeted strategies to enhance vaccine effectiveness. This may include novel adjuvants or combination therapies to modulate specific memory B-cell subsets, facilitating their activation through T-cell stimulation and thereby promoting a more robust and durable immune response. Overall, the data presented in this study provided valuable insights into the differential immune memory B-cell responses following influenza virus vaccination. These findings pave the way for future research to further unravel the complexities of vaccine-induced memory B-cells and ultimately improve vaccination strategies against influenza virus infection.

This study presents limitations that should be considered. One major limitation is the sample size, which restricts the statistical power of our analyses. Although we explored the potential impact of age on immune responses by performing an age-stratified analysis, the small number of participants in each group limited our ability to draw meaningful conclusions. Additionally, as supported by existing healthy aging study, changes in the immune system tend to be more pronounced after 70 years of age, with little significant alteration observed in individuals below this threshold (38). Since our study population spans an age range of 23–66 years, we believe the potential influence of age on our findings is likely minimal. Additionally, the study did not account for pre-existing immunity among participants, which could significantly impact the humoral responses observed post-vaccination. Future studies with larger sample sizes are warranted to further elucidate these factors and their effects on vaccine-induced immune responses.

## Data Availability

The original contributions presented in the study are included in the article/**Supplementary Material**. Further inquiries can be directed to the corresponding author/s.
