# Supplementary figures and images for: B-cell dynamics underlying poor response upon split-inactivated influenza virus vaccination
Source: Front Immunol. 2024 Nov 20;15:1481910. doi: 10.3389/fimmu.2024.1481910 (PMC11614812; doi:10.3389/fimmu.2024.1481910)

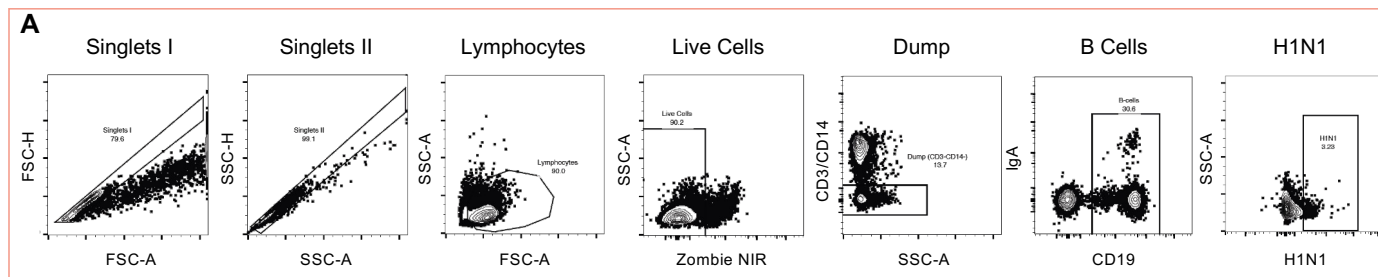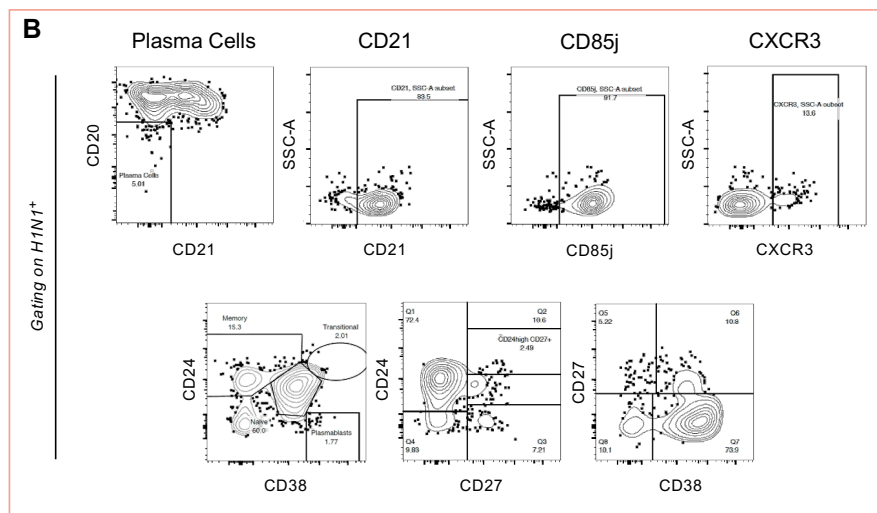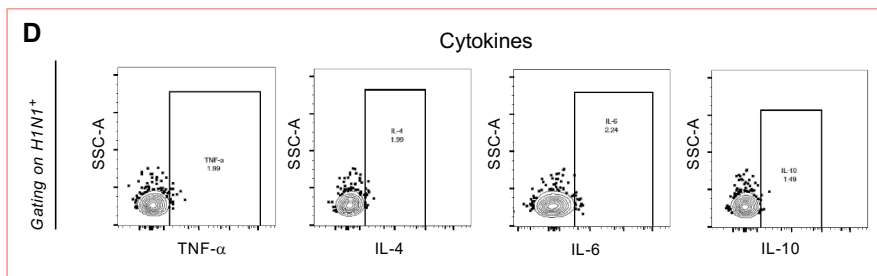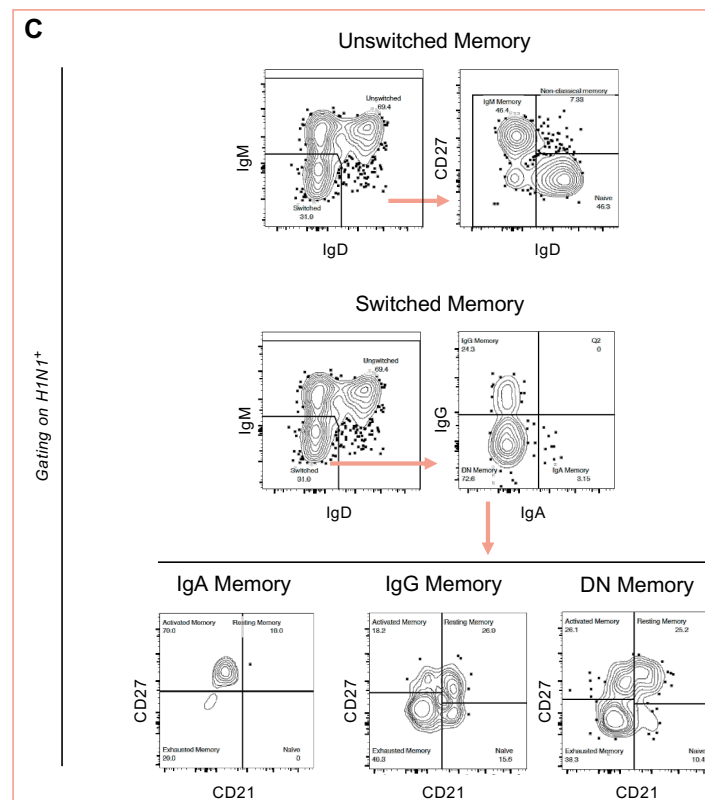

Supplementary Figure 1

Supplement: Supplementary file 1 [file DataSheet1.pdf]
